# EBMT/ESID inborn errors working party guidelines for hematopoietic stem cell transplantation for inborn errors of immunity

**DOI:** 10.1038/s41409-021-01378-8

**Published:** 2021-07-05

**Authors:** A. C. Lankester, M. H. Albert, C. Booth, A. R. Gennery, T. Güngör, M. Hönig, E. C. Morris, D. Moshous, B. Neven, A. Schulz, M. Slatter, P. Veys, on behalf of the Inborn Errors Working Party of the European Society for Blood and Marrow Transplantation and the European Society for Immune Deficiencies, and European Reference Network on Rare Primary Immunodeficiency Autoinflammatory Autoimmune diseases (RITA)

**Affiliations:** 1 Willem-Alexander Children’s Hospital, Pediatric Stem Cell Transplantation Program, Leiden University Medical Center, Leiden, The Netherlands; 2 Department of Pediatrics, Dr. von Hauner Children’s Hospital, University Hospital, LMU, Munich, Germany; 3 Great Ormond Street (GOS) Hospital for Children NHS Foundation Trust and University College London GOS Institute of Child Health, London, UK; 4 Translational and Clinical Research Institute, Newcastle University, and Paediatric Haematopoietic Stem Cell Transplant Unit, Great North Children’s Hospital, Newcastle upon Tyne, UK; 5 Department of Hematology, Oncology, Immunology, Gene-therapy and Stem Cell Transplantation, and Children’s Research Center (CRC), University Children’s Hospital, ZurichZurich, Switzerland; 6 Department of Pediatrics, University Medical Center Ulm, Ulm, Germany; 7 Institute of Immunity and Transplantation, University College London Hospitals NHS FT and Royal Free London Hospitals NHS FT, London, UK; 8 Unité d’immune-Hematologie et Rhumatologie Pédiatrique, Hôpital Necker Enfants Malades, Assistance Publique-Hôpitaux de Paris, Paris, France; 9 Université de Paris, Paris, France; 10 Institut Imagine, INSERM UMR1163, Laboratory of genomic Dynamics in the Immune system, Paris, France; 11 Institut Imagine, INSERM UMR1163, Laboratory of Immunogenetics of Pediatric Autoimmune Diseases, Paris, France

**Keywords:** Immunology, Immunological disorders

## Introduction

Inborn errors of immunity (IEI) are a group of rare heterogeneous diseases. Currently, more than 400 monogenetic IEI have been identified and increasingly a genetic diagnosis can be made in patients with an immune deficiency disorder [1]. Patients may present with a variety of clinical symptoms including a broad spectrum of infections, inflammatory manifestations, auto-immune phenomena and malignant diseases. Treatment by hematopoietic stem cell transplantation (HSCT) is increasingly successful [2–10] and the joint EBMT/ESID Inborn Errors Working Party (IEWP) has played a pivotal role designing and developing common HSCT guidelines, which have contributed to this success.

The wide clinical heterogeneity of patients, together with the fact that outcome data are based on observational rather than prospective studies, means that it is not yet possible to recommend strictly defined protocols for transplanting IEI patients. The current guidelines provide recommendations based on published data, center experience and expert opinions. Whenever possible, the individual transplant protocol should follow these guidelines, but modifications may be necessary according to the particular variant of the IEI and/or the patient’s clinical condition. For all these reasons the IEWP strongly recommends that all patients with primary immunodeficiency are transplanted in an experienced center that regularly transplants such patients, and also actively participates in the IEWP, as only in this way continuous improvement in outcomes can be achieved.

The prognosis of survival for some patients with IEI extends for years and even decades with conservative therapy alone. In those, the decision in favor or against HSCT or other cellular therapies can be extremely challenging. This decision needs to consider multiple factors such as clinical presentation, past and current infections, immunophenotype, genotype, autoimmune manifestations, current and anticipated future organ damage, family history and family experience with the disease, psychological and social factors such as quality of life and fertility, and informed consent not only of caregivers but also patients themselves. In case of a decision for a conservative treatment strategy, this should be re-evaluated on a regular basis by a team, which is informed about and experienced in all currently available therapeutic options. Centers are strongly advised to register their transplanted patients in the EBMT, ESID, and SCETIDE registries, which will allow continuous evaluation of the outcomes in transplanted IEI patients treated in line with IEWP guidelines.

Patients with IEI frequently present with or develop autoimmune or inflammatory complications eg, autoimmune cytopenias and inflammatory bowel disease as their sole clinical phenotype [11]. In recent years, monogenetic defects are increasingly identified in patients with primary immune regulation disorders (PIRD [12]). Awareness of the possibility that a monogenetic IEI may be the underlying defect in patients with aforementioned disease manifestations is pivotal in their clinical management and may provide the rationale for allogeneic HSCT as a curative approach [13].

In recent years, stem cell gene addition therapy (GT) has been explored for a limited number of IEI, including ADA-SCID, X-linked SCID, XL-CGD, and WAS. A retroviral ADA GT product (Strimvelis®) is licensed by the European Medicines Agency, and recently excellent results have been reported with lentiviral-based ADA GT [14]. There are ongoing clinical studies in a variety of other IEI [15]. GT offers the potential advantage of avoiding the negative consequences of alloreactivity (GVHD), but concerns remain about the curative potential of a mixed chimeric state in non-SCID IEI, which is inherent to current GT approaches, and the possible risk for insertional mutagenesis (although no vector related adverse events have been reported with lentiviral vectors). However, in the absence of comparative studies it is extremely difficult to make firm recommendations on the hierarchial position of GT in comparison to conventional HSCT. It has to be considered that: (a) long-term safety and efficacy data of GT are still limited, and (b) comparing outcome data from prospective single- or oligocentre studies (as is the case for GT) with retrospective multi-center studies (as most of the evidence for HSCT) does not meet the scientific standard and is therefore suboptimal. Currently, participation in a GT study may be considered for patients lacking a matched donor and able to travel to a respective study center.

The IEWP guidelines are reviewed periodically and retrospective studies are regularly performed on behalf of IEWP to evaluate and compare clinical outcomes of patients with specific disease entities treated according to these guidelines [3, 9, 16]. These studies are instrumental to periodically revise and update the guidelines for specific conditions.

## Pretransplant considerations

While until 15–20 years ago, myeloablative conditioning with (oral) busulfan and cyclophosphamide was the norm, over the years, extensive experience has been obtained with a number of different, and increasingly less toxic, conditioning regimens [9, 17–19]. Moreover, therapeutic drug monitoring has resulted in improved outcome in busulfan-based regimens [20]. The guidelines are recommended based on reported data, taking into consideration both patient and disease-specific parameters as well as center differences in experience with and availability of some of these drugs.

The aim and rationale to recommend the IEWP guidelines are as follows:

1. By limiting the number of recommended protocols, there will be less variation between centers in the treatment of these rare diseases.
2. If centers use specific protocols as defined, we will be able to gather retrospective data on the performance of a specific protocol in treating these conditions.
3. We also recognize that for smaller or less experienced centers this guidance is important to optimize patient management, and by making these guidelines available on the EBMT and ESID websites the information is readily available.

We have therefore agreed on six protocols, labeled A–F, which are recommended for the majority of IEI (Table 1). Specific details of these protocols are available in Appendix 1. Disease category specific considerations are provided in section C. Given recent developments in the field of haplo-identical/mismatched donor HSCT, specific recommendations are provided for the two most commonly used approaches.

**Table 1:** Conditioning regimens.


A				Busulfan i.v (AUC = 85–95 mg*h/L) Fludarabine (160 mg/m^2^)
B				Treosulfan (30–42 g/m2) Fludarabine (150–160 mg/m^2^) Thiotepa (8–10 mg/kg)
C			Busulfan i.v. (AUC = 60–70 mg*h/L) Fludarabine (160–180 mg/m^2^)	
D			Treosulfan (30–42 g/m^2^) Fludarabine (150–160 mg/m^2^)	
E		Fludarabine (150–160 mg/m^2^) Melphalan (140 mg/m^2^)		
F	Fludarabine (150 mg/m^2^) Cyclophosphamide (20–40 mg/kg)			

Protocol A and B: These are recommended for patients without severe preexisting organ damage and non-SCID diseases where a complete donor chimerism is desired for optimal disease correction.

Protocols C and D: These are recommended for patients with preexisting organ damage and/or diseases where engraftment has been shown to reliably occur with reduced intensity conditioning. Mixed donor chimerism is more likely to occur compared to protocols A and B.

Protocol E: This may be best suited for patients with preexisting organ damage and/or diseases where full myeloid engraftment is not absolutely required. Higher degrees of chimerism can be achieved when using PBSC. DLI may be required in case of mixed chimerism.

Protocol F: To avoid organ toxicity this regimen is only recommended for patients with DNA repair/radio-sensitivity disorders (except Artemis deficiency) in which alkylating agents are used in low dose.

We strongly recommend that, when these protocols are used, transplant centers adhere to them as much as possible in terms of dosing and schedule since only then, can meaningful data be generated and collected over time via the EBMT, ESID, and SCETIDE registries.

### Specific recommendations on HLA-typing, donor hierarchy, conditioning agents, and stem cell dose

#### HLA-typing

High resolution, allele level HLA-A, -B, -C and -DRB1, -DQB1 typing is mandatory for all donors (except unrelated CB, see below). For unrelated donors, allele-level matching at all 10 loci is the gold standard. While some centers would also consider a 9/10 as a matched unrelated donor (MUD), others would call it a mismatched unrelated donor (MMUD) and prefer to apply the approaches recommended below for haploidentical transplants with such a donor (vide infra). Any donor with less than 9/10 match should be considered mismatched.

#### Donor hierarchy

In the last decade HSCT ouctomes in IEI have steadily improved for the different donor types due to improved HLA typing technology and therefore donor selection, better management of HSCT complications as well as improved supportive care. Outcomes obtained with MUD have increasingly improved and are approaching now survival rates similar to those of matched sibling donors (MSD) [3, 10, 16]. However, the higher rates of HSCT complications with MUD still justify the position of HLA identical siblings as the first donor choice in most cases. That notwithstanding, given the genetic basis of IEI, family donor screening should check whether a candidate donor is affected by, or in some diseases a carrier of, the same genetic defect, especially in diseases with a late or variable onset of clinical presentation [21–23].

In recent years, HSCT performed with mismatched family and unrelated donors have demonstrated improved and encouraging results [24–27], and should be considered a reasonable alternative in the absence of a matched donor. However, higher rates of complications (e.g., infections, graft failure) may occur in these transplants. Therefore, and even more than in HSCT for IEI in general, transplantation with mismatched donor should only be performed in centers with experience in these procedures in IEI patients. These alternative donors are increasingly considered at an earlier stage to avoid postponement of transplantation and thus the risk of performing the transplant later in patients with an unfavorable risk profile.

#### Busulfan

To optimize busulfan exposure therapeutic drug monitoring (TDM) is mandatory. In these guidelines two busulfan regimens are recommended: Busulfan protocol A (myeloablative) and protocol C (reduced intensity).

Busulfan is used as an i.v. formulation. Busulfan may be administered once daily, twice daily, or four times daily as per institutional standard (see Table 2). The initial busulfan dose is based on weight or body surface area and subsequent doses are based on TDM [20] Repeated TDM is recommended in case the first dose adjustment is >10% and in infants [28]. Alternative model-based approaches for busulfan dosing have been reported recently [29].

**Table 2:** Busulfan dosing scheme for protocol A and C.

Body weight (kg)	Dose (mg/kg)^a^ 4 days^b^
Once daily (3 h-infusion)	
3–15	5.1
15–20	4.9
25–50	4.1
50–75	3.3
75–100	2.7
Twice daily (3 h-infusion)	
3–15	2.5
15–20	2.4
25–50	2.1
50–75	1.6
75–100	1.3
Four times daily (2 h-infusion)	
3–15	1.3
15–20	1.2
25–50	1.0
50–75	0.8
75–100	0.7

^a^TDM doses of Bu are given at same rate (in mg/h) as initial dose, so may not be over 3 h.

For full myeloablative dose (i.e., protocol A) aim for a cumulative Busulfan dose of:

AUC 85–95 mg/L × h (target 90) = 85,000–95,000 ng/ml × h = 20,706–23,180 mmol min

For reduced intensity myeloablative dose (i.e., protocol C) aim for a cumulative Busulfan dose of:

AUC 60–70 mg/L × h (target 65) = 60,000–70,000 ng/ml × h = 14,616–17,052 mmol min

^b^The full myeloablative dose (protocol A) is given in 4 days, whereas the reduced intensity myeloablative dose (protocol C) may be administered in 3–4 days (see also Appendix).

#### Treosulfan

Treosulfan is known for both its myeloablative and immune suppressive activity, and its favorable toxicity profile [18, 30–32]. Similar overall survival and outcome have been reported with myeloablative busulfan and treosulfan-based regimens in several malignant and non-malignant diseases. Still, the predictability to reach full donor chimerism is less in the case of treosulfan compared to high AUC targeted busulfan regimens, which may be relevant in those diseases where full donor chimerism is preferred. Although treosulfan exposure in vivo has been linked to clinical outcome parameters, pharmacological studies do not yet provide convincing support for therapeutic drug monitoring as a general approach to guide individual dosing [18, 33, 34]. Therefore, treosulfan dose recommendations currently remain based on body surface area (3 × 10 g/m^2^ in <0.5 m^2^, 3 × 12 g/m^2^ in 0.5–1.0 m^2^, 3 × 14 g/m^2^ in ≥1 m^2^). In these guidelines two treosulfan-based regimens are proposed, B (myeloablative) and D (reduced intensity).

#### Fludarabine

Fludarabine is primarily a lymphodepleting agent that is commonly used in combination with either busulfan or treosulfan instead of cyclophosphamide. Recommendations on dosing are based on body surface area (4 × 40 mg/m^2^; 5–6 × 30 mg/m^2^). In infants, Fludarabine dosing may be more accurate on a body weight basis although well-defined guidelines are currently lacking. PK-guided dosing has been proposed to result in improved outcome but these results require confirmation in larger studies before specific recommendations can be made [35–37].

#### Thiotepa

Thiotepa is an alkylating drug which is often combined with either treosulfan and fludarabine as a myeloablative regimen. Thiotepa may add myeloablative activity and also has the potential to cross the blood–brain barrier which may beneficial in diseases with CNS involvement (e.g., HLH) [38, 39].

#### Cyclophosphamide

Whereas cyclophosphamide has been largely replaced by fludarabine when used in combination with busulfan or treosulfan, it is recommended at a significantly reduced dose in protocols used for patients with Fanconi anemia and DNA-repair disorders [40, 41]. In addition, cyclophosphamide is being used post-transplant (PT-Cy) as in vivo T-cell depletion strategy, particularly in mismatched donor transplantation (vide infra).

#### Melphalan

Increasing recognition of the significant toxicities associated with historical use of busulfan and cyclophosphamide, particularly in very young infants, and those with preexisting end organ damage, led to the adoption of more immunosuppressive, rather than fully myeloablative regimens, with fludarabine and melphalan (protocol E). The results, principally in those with significant preexisting co-morbidities, were striking with significantly improved early survival [42]. However, donor chimerism was not always optimal, there was a high incidence of late viral re-activation, and late onset acute GvHD. Furthermore, toxicities in infants <1 year of age remained significant. In particular, melphalan has been associated with cardiac toxicities [43]. Favorable overall survival was reported in in haemophagocytic lymphohistiocytosis (HLH) patients, although DLI or a second transplant procedure were frequently required to overcome graft failure [44]. Moderate results were reported in X-linked inhibitor of apoptosis protein (XIAP) deficiency [45]. It has also been used successfully in adults with IEI [46].

#### ATG/ATLG and alemtuzumab

Serotherapy is an essential element in most conditioning regimens to prevent graft rejection as well as GvHD. Serotherapy is mandatory in all unrelated and mismatched family donor transplants. However, it may also be an option in selected HLA identical family donor transplants, particularly in diseases with an inflammatory component [19]. Different biological products may be used to achieve these goals: polyclonal rabbit anti-thymocyte globulin (ATG/Thymoglobuline^®^) and anti-T lymphocyte globulin (ATLG/Grafalon^®^), and CD52 monoclonal antibody alemtuzumab. Recent studies in mixed populations of malignant as well as non-malignant diseases have indicated that ATG and alemtuzumab exposure following standard dosing is highly variable with an impact on clinical outcome. Moreover, body weight and lymphocyte numbers at start of ATG treatment have been reported as important parameters [47–49]. Although these factors are probably equally important in the highly heterogenous population of patients with inborn errors of immunity, it remains difficult to provide a specific and indivualized recommendation on dose and timing in particular based on an extrapolation of current knowledge and published data in other disease categories [50]. ATG/ATLG and alemtuzumab PK/PD studies are ongoing involving patients with inborn errors of immunity that may eventually result in more specific and individualized recommendations including PK guided dosing. Considering aforementioned limitations, current recommendations are made based on available, albeit limited, published data and common practice in experienced centers. The recommended dose ranges for unmanipulated grafts are: Thymoglobulin 5–10 mg/kg (total dose), Grafalon 15–30 mg/kg (total dose) and alemtuzumab 0.6–1.0 mg/kg (total dose) starting at day-8/-7. To avoid overexposure of serotherapy resulting in prolonged lympho-/immunodepleting activity following infusion of the graft, serotherapy administration may be scheduled more distal to the graft. The latter is particularly important in lymphopenic individuals and when using cord blood grafts and may benefit from TDM [50, 51]. Based on published data, specific recommendations on serotherapy are provided for haplo-identical/mismatched donor HSCTs in the context of the TCR α/β depletion approach and the T replete marrow-PT-Cy approach (Table 3).

**Table 3:** Haploidentical HSCT platforms.

	TCR α/β [26, 27, 54]	PT-Cy [24, 25]	CD34 positive selection (*only recommended for some SCID transplants*)
Protocols	A, B, C, D	A, B, C, D	C, D
Graft	TCR α/β - CD19 depleted PBSC	unmanipulated bone marrow (1st choice) or PBSC^a^ (2nd choice)	CD34 positive selected PBSC
Cell dose	10-20 x 10e^6^ CD34/kg	3–5 × 10e^8^ TNC/kg	10–20 × 10e^6^ CD34/kg
Serotherapy	ATG Grafalon: 3 × 4 mg/kg (d-4 to -2)^b^ Rituximab: 200 mg/m2 (d-1)	Alemtuzumab: 2 × 0.2 mg/kg (d-10 to -9) If Alemtuzumab is not available: ATG Thymoglobuline 3 × 2.5 mg/kg (d-10 to -8)^c^	None
GVHD prophylaxis	If αβ T cells in graft ≥10e5/kg: add CSA	Cyclophosphamide 50 mg/kg on d + 3 and d + 4 Tacrolimus or CSA from d + 5 until at least d + 100 MMF from d + 5 to d + 35	none

^a^In case PBSC are used, higher rates of cGVHD can be expected and additional or prolonged GvHD prophylaxis may be considered [101].

^b^In case there is no access to ATG-Grafalon alternative serotherapy approaches (i.c. thymoglobulin) may be considered although published recommendations on optimal dose and timing are currently unavailable.

^c^In case of ATG in PT-Cy protocol additional Rituximab may be considered.

#### Graft versus host prophylaxis

Several GvHD prophylaxis regimens with comparable clinical efficacy are commonly used. These regimens are often based on local experience and take preexisting co-morbidity, which frequently occurs in IEI patients, into consideration. Therefore, we recommend to use GvHD prophylaxis with a calcineurin inhibitor plus a second agent (MTX or MMF) in accordance with institutional guidelines for unmanipulated grafts. In case unmanipulated PBSC are used from MUD, higher rates of cGVHD can be expected and prolonged GvHD prophylaxis may be considered. In mismatched family (MMFD) and MMUD, graft manipulation (either ex vivo or in vivo) should be strongly considered depending on the degree of mismatch, to limit the risk for GvHD, using the TCR α/β depletion or PT-Cy approach, respectively (vide infra).

#### Stem cell dose

In case of unmanipulated BM grafts in matched family or unrelated donors the recommended cell dose is 2–4 × 10e^8^ TNC/kg. In case of T replete PBSC grafts from matched donors the recommended CD34^+^ cell dose is 5–8 × 10^6^/kg. In case of higher doses, the T cell dose should not exceed 3–5 × 10e8 CD3^+^ T cells/kg in order to limit the risk for GvHD [52]. For MMUD/MMFD donors specific recommendations are provided in Table 3.

In recent years, the number of unrelated cord blood transplants has decreased due to new methods used for haploidentical transplants. However, in some centers cords are the preferred stem cell source when an alternative donor is required. Units should be preferentially selected from cord blood banks that have achieved FACT-NETCORD accreditation to ensure the quality of the products chosen. Unit selection is based on HLA matching and cryopreserved total nucleated cell (TNC) ± CD34^+^ doses. HLA-A, -B, -C, and -DRB1 typing should be performed at the allelic level. All units must be ≥7/8 matched to the recipient. Priority is given to an 8/8 matched single unit graft with a TNC ≥ 3.0 × 10e^7^/kg followed by 7/8 matched single unit graft with a TNC ≥ 5.0 × 10e^7^/kg after thawing. CD34 + counts are not standardized but ideally should be >1.7 × 10e^5^/kg after thawing [53].

#### Recommendations for haploidentical HSCT and mismatched unrelated donors

Based on published data and the experience from participating IEWP centers, two recommendations are being made for transplantation with MMFD and MMUD based on the TCR α/β depletion approach and the T replete marrow-PT-Cy approach [24–27, 54] (Table 3). The choice of the conditioning regimen (A, B, C, D) will depend on the type of disease and the clinical condition of the patient as further discussed in the disease-specific paragraphs. Other approaches including irradiation-containing regimens have been used with success, nevertheless when possible irradiation free regimens are preferred [55, 56]. CD34 positive selection is only recommended as an option in selected SCID patients.

## Disease category specific recommendations

In this section, recommendations are provided on conditioning with the aim to be as uniform as possible for the different disease categories unless there is a very specific, evidence-based reason to have a distinct approach. The latter seems particularly the case for selected SCID patients and patients with a radiosensitivity disorder.

### Severe combined immunodeficiency

#### Background

Severe combined immune deficiencies (SCID) are a heterogeneous group of inherited disorders characterized by impaired T-lymphocyte differentiation. All underlying genes intrinsically affect crucial steps in the T-cell maturation. Typical cases are characterized by the absence of autologous T lymphocytes (<300/µl) and deficient T-lymphocyte proliferation while B lymphocytes and NK cells lymphopoeisis can be variably affected. SCID are phenotypically divided in B+/B- and NK+/NK- SCID and, in typical cases, correspond to the genetic entities as indicated (examples):

T^−^B^−^NK^−^: *ADA, PNP, AK2*

T^−^B^+^NK^−^: *JAK3, IL2RG*

T^−^B^−^NK^+^: *RAG 1/2, DCLRE1C, LIG4, PRKDC, NHEJ1 (*cernunnos/XLF*)*

T^-^B^+^NK^+^: *IL7RA, CD3 (D,E,Z), CD45, CORO1A*

Hypomorphic mutations (with residual protein-expression and function) in the indicated genes typically responsible for SCID can result in Omenn’s syndrome or a less severe immunological phenotype with higher number of autologous lymphocytes which will be discussed hereafter in “Hypomorphic SCID and Omenn Syndrome”. SCID related to DNA Ligase 4, Cernunnos- XLF, Nijmegen Breakage Syndrome will be discussed in the section “Radiosensitivity Disorders”.

If not detected in a neonatal screening program or with an informative family history, patients with SCID present with serious, recurrent and potentially life-threatening infections within the first year of life. Infections are caused by a broad spectrum of viral, fungal, bacterial, and opportunistic infectious agents. Because of the strong impact of early diagnosis on prognosis, neonatal screening has been implemented in several countries. However, not all patients who are at risk for such a presentation will be detected by screening programs based on the determination of T cell receptor excision circles levels. Patients need to be managed in experienced and dedicated centers. Ongoing infections need to be aggressively treated; immunoglobulin replacement therapy and PJP prophylaxis need to be given, while nutritional status will need specific attention in many cases. Patients need to be isolated while preparing HSCT with regular infectious screening to allow timely treatment in case of infection. Breast-feeding from a CMV positive mother should be avoided, and live vaccines are forbidden. If the patient has received Bacille Calmette Guerin (BCG) immunization before diagnosis, prophylactic treatment with two antimycobacterial drugs is recommended in the absence of symptoms while the therapeutic administration of four drugs has been recommended in case of BCGitis [57]. Blood products need to be irradiated before transfusion to avoid GVHD.

Once the diagnosis of SCID has been established, there is an urgent need for identification of a suitable donor. HSCT from a geno-identical/matched sibling remains the gold standard; other matched related donors (MRD) can be considered likewise. In the absence of matched family donors, the choice will depend on availability of a 10/10 MUD. If the delay to donor recruitment is not compatible with the clinical status of the patient or in the absence of such donors, HSCT from a haploidentical family donor or a mismatched unrelated cord blood will be the preferred choice. A quick genetic diagnosis is strongly desired and may be helpful to devise the best HSCT approach especially in radiosensitive disorders.

With regard to the stem cell source, most centers would choose bone marrow for unmanipulated grafts but would prefer peripheral blood stem cells in case graft manipulations such as TCRαβ/B-cell depletion or CD34 + stem cell selection are needed. Currently, experience with the haplo PT-Cy approach when using mismatched donors in SCID is scarce but may be considered in the absence of aforementioned treatment options [58].

Choice of conditioning regimen will be mainly based on donor type and SCID phenotype, as well as genotype if available [59] (Table 4). Full myeloid chimerism is not mandatory but a degree of myeloid engraftment will help to sustain B cell reconstitution and long-term thymic output. Clinical status of the patient may be balanced with intensity of myeloablation in some circumstances. In case of a life-threatening infection, no tolerance for toxicity, and the availability of a genoidentical matched family donor, the infusion of an unmanipulated T-cell replete graft opens the opportunity to establish a functional T-cell system from mature donor-derived T lymphocytes, which proliferate and expand in the recipient within days and are able to control infections. In case of genoidentical donors this can and has been successfully achieved without any GvHD-prophylaxis. The potential benefits of this approach without myelosuppressive agents (myeloid engraftment may occur due to a graft versus marrow effect) and avoidance of side effects, have to be balanced against the risk of GvHD and the potentially prolonged immunosuppression that is needed for GvHD treatment.

**Table 4:** Donor-and genotype-related conditioning regimens in SCID.

	MSD/MRD^c^	MUD/MMUD/MMFD^c^
JAK3, IL2Rγ (T^-^B^+^NK^-^)	No^a^/C/D	C/D
RAG1/2, DCLRE1C^b^ (T^-^B^-^NK^+^)	C/D	C/D
IL7Rα, CD3 δ,ε,ζ, CD45 (T^-^, B^+^, NK^+^)	No^a^/C/D	C/D
ADA	No^a^/C/D	C/D
AK2	C/D	C/D

^a^No: no conditioning.

^b^See specific comments on DCLRE1C in the text.

^c^MSD: matched sibling donor, MRD: matched related donor, MUD: matched unrelated donor, MMUD: mismatched unrelated, MMFD: mismatched family donor.

**In T-B** + **NK- SCID**, the absence of NK cells and the early block in T cell differentiation abolish the risk of rejection, allowing T cell reconstitution in the absence of myeloablation, leading to split chimerism with only T cells from the donor. However, B cell reconstitution will not be restored in most patients, requiring lifelong immunoglobulin replacement. In addition, thymopoiesis will decrease over time leading to progressive decline of naïve T cells counts, possibly associated with clinical events in the long term. HSCT from genoidentical donors is the exception to this rule with expected (but not guaranteed) B cell reconstitution and long-term thymic output, allowing consideration of HSCT without myeloablation.

**In T-B-NK** + **SCID**, mainly related to defective VDJ recombination, alloreactivity from the recipient NK cells and competition for thymic niches (between double negative thymocytes from the recipient and precursors from the donor) will require some degree of myeloablation at least when a mismatched donor is used and to achieve B cell engraftment.

Patients with T-B-NK + SCID related to *DCLRE1C (Artemis)* need specific attention. It was shown that the use of alkylating agents in the conditioning regimen was associated with long-term clinical problems such as microdonthia or absence of definitive teeth, shorter height and mild renal impairment in teenage years [60]. These long-term problems need to be balanced with the risk of graft failure, especially if the HSCT is performed from a mismatched donor in the absence of myeloablation.

In **T-B** + **NK** + **SCID**, B cells are not primarily affected. Therefore, strictly speaking, B-cell and myeloid engraftment may not be necessary for B-cell function. However, using the same reasoning for this group as for the others, T-cell function with stable myeloid engraftment may lead to better long-term overall immune function in comparison to split chimerism.

*In ADA-deficiency* three therapeutic options are available including Enzyme replacement Therapy (ERT) and gammaretroviral gene therapy, which was licensed in 2016, beyond the option of an allogeneic HSCT. ERT is an important and readily available tool for the initial treatment of seriously sick patients and for bridging to cellular therapies, but is not recommended for long-term treatment. If a HLA-identical family donor is available, most centers would choose infusion of a graft without prior conditioning or GvHD prophylaxis. This strategy has been demonstrated to lead to stable immune functions (including B cells) and permanent cure in most patients, although use of myeloablative conditioning will improve B cell engraftment.

In the studies leading to the approval of gene therapy for patients with ADA-SCID overall survival was 100% [61]. Even though there is limited experience in ADA-deficient patients for MUD transplants and a less favorable outcome for historic patients transplanted from haploidentical donors, these options need to be considered if gene therapy is not available or not feasible in the current setting. Gene therapy for other SCID entities is performed in several clinical trials executed in expert centers [15]. These studies have specific inclusion criteria, which are beyond the scope of these guidelines.

In summary, conditioning increases the likelihood of myeloid engraftment, thymic output and independence from Ig in SCID patients. Therefore conditioning is recommended as a default position in most cases. If a patient is not deemed fit to tolerate condition, an unconditioned rescue infusion may be performed, with a variable risk of absent B cell reconstitution, a decline in thymopoesis overtime, and high risk of graft failure in T-B-NK + SCID. In these cases unconditioned infusion is associated with the expectation that the patient will undergo a second, conditioned, procedure when they have recovered without evidence of durable immune reconstitution. In patients with AK2 deficiency (Reticular Dysgenesis), transplantation without conditioning is associated with a high risk of primary graft failure especially in haploidentical transplantation with in vitro T-cell depletion of the graft [62].

For patients transplanted without conditioning from MUDs, serotherapy is highly recommended to reduce the risk for GvHD [63].

As there is limited experience in newborns with regard to pharmacokinetics, toxicity and tolerance of drugs used for conditioning, conditioned HSCT iis not generally recommended before 6–8 weeks of age [59].

Maternal T-cells need to be controlled before transplantation because of clinical symptoms (exanthema, diarrhea, liver disease), potential organ damage and the risk of graft rejection. This can be managed by early serotherapy, immunosuppressive drugs such as CSA or Tacrolimus, and immunosuppressive components in the conditioning regimen (Fludarabine).

### Hypomorphic SCID and Omenn syndrome

Hypomorphic SCID and Omenn syndrome are both due to hypomorphic mutations (with residual expression and activity) in genes associated with typical SCID [64]. Mutations in *RAG1* and *RAG2* are the most frequently associated with this broad range of phenotypes [65]. In the absence of hypomorphic mutations in SCID genes, the diagnosis of Di George syndrome should be considered, particularly because of the therapeutic impact. Omenn syndrome is characterized by early onset generalized skin rash, alopecia, hepatosplenomegaly, polyadenopathy, hypereosinophilia and raised IgE. This condition is related to oligoclonal expansion of activated autologous T cells that infiltrate the target organs, mainly the skin and the gut, but liver and lung can also be affected. Failure to thrive, poor nutritional status and diarrhea are frequent. Incidence of bacterial sepsis is high due to altered skin and gut barriers. Immunosuppression before HSCT is frequently required to control skin and gastrointestinal infiltration and inflammation. To avoid prolonged deleterious treatment with steroids, cyclosporine A or serotherapy with alemtuzumab can be proposed. Ongoing inflammation at the time of HSCT should be avoided to prevent activation of donor T cells that may exacerbate the risk of GVHD. Conditioning is mandatory in Omenn syndrome and, as in SCID, protocol C or D is recommended. HSCT in Omenn patients is associated with a high rate of endothelial toxicites and defibrotide prophylaxis can be considered.

Patients with hypomorphic SCID are clinically and immunologically very heterogenous. Clinical onset may be delayed and patients may present with autoimmunity and granuloma. Residual immunity is significant in these patients that belong to the heterogenous category of CIDs. Myeloablative but reduced toxicity conditionning (protocol C/D) is usually prefered but comorbities may prevent the use of full myeloablation.

### Radiosensitivity disorders

Patients with combined immunodeficiencies due to radiosensitive disorders such as DNA ligase 4 deficiency, Cernunnos-XLF deficiency or Nijmegen breakage syndrome may be detected by newborn screening for SCID, or present with immunodeficiency, autoimmunity, myelodysplasia or malignancy, particularly leukemia or lymphoma, and may require transplantation [41]. There are too few data available to recommend HSCT as routine standard of care. As many of the conditioning regimens are particularly damaging to DNA, less toxic regimens are required to successfully treat these patients. Radiotherapy should be avoided due to catastrophic toxicity [66]. Conventional doses of alkylating agents are generally poorly tolerated, often leading to multi-organ failure and early transplant-related mortality. It should be noted that, in particular, the long term outcome of these patients following HSCT has yet to be determined and indication for HSCT should be set only on a case-by-case basis after careful consideration of risks and benefits and possibly in consultation with other centers that have transplanted these patients. One large multicenter study looked at outcomes of transplant for patients with DNA ligase 4 deficiency (36 patients), Cernunnos-XLF deficiency (17 patients) and Nijmegen breakage syndrome (26 patients) [40]. Best survival was achieved when patients received a modified Fanconi-type conditioning regimen—we recommend protocol F, but others have been used. Careful longterm follow up is recommended, particularly looking for occurrence of secondary malignancies, although none are reported to date.

### Ataxia-Telangiectasia

There are few reports of HSCT for patients with Ataxia-Telangiectasia. The same issues regarding sensitivity to radiation and alkylating agents applies to these patients as to those with DNA ligase 4 deficiency, Cernunnos-XLF deficiency and Nijmegen breakage syndrome. However, given the progressive neurological deterioration that A-T patients experience, there is currently no place for routine HSCT for these patients. As the majority of those being considered for HSCT will have lympho-hematological malignancy requiring chemotherapy, HSCT cannot be recommended routinely for these patients. Treatment schemes for malignancies need to be carefully adapted on a case-by-case basis taking into consideration alternate immunotherapy based options whenever possible. The optimal management of those picked up by newborn SCID screening has yet to be determined.

### Combined immunodeficiencies

#### Background

Combined immunodeficiency (CID) represents a category of IEI which, compared to SCID, is characterized by a less profound quantitative or functional T cell defect, which is often accompanied by a B cell defect. CID may present as an isolated immune disorder (e.g., CD40 Ligand deficiency, Bare Lymphocyte Syndrome, CD27-CD70 deficiency, DOCK 8 deficiency) or as immune disorders with associated or syndromic features (e.g., Wiskott Aldrich syndrome (WAS), autosomal dominant anhidrotic ectodermal dysplasia with immune deficiency (AD EDA-ID) [3, 8, 67–69]. Occasionally, also radiosensitivity disorders as described above may present as CID [12]. The broad spectrum of genetic defects causing CID is further complicated by heterogenous clinical presentations, thus creating challenges regarding the indication for and timing of SCT [70, 71].

#### Preferred regimen

CID patients have impaired, but residual T cell immunity and often intact myelopoiesis. To achieve sustainable donor stem cell engraftment and T cell immunity, protocol A or B is usually preferred. This is especially true in diseases where mixed chimerism is associated with inferior outcome as for example in WAS [3, 67, 72]. However, preexisting infectious and non-infectious comorbidities or older age may increase the risk of transplant-related mortality and therefore often preclude the use of a fully myeloablative approach. Particularly in (older) patients with comorbidity, protocols C and D (and to a lesser extent E) have been demonstrated to result in favorable outcomes for both OS and EFS [46, 73]. The impact of comorbidity on transplant outcome is also reflected in the observation that in various single disease studies younger age at transplant as well as shorter time from diagnosis to transplant, are correlated with better overall and event-free survival [3, 7]. In order to address the fact that the most appropriate conditioning regimen may also be disease specific, a recently published review series on behalf of IEWP covering HSCT in different CID disease identities provides more disease-specific recommendations [8, 67, 69, 71].

#### Specific considerations

Control of, or—even better—prevention of infectious complications prior to SCT and performing HSCT prior to development of organ damage, significant autoimmunity, chronic (EBV) infection or malignancy will result in less SCT-related complications and superior outcome.

#### Alternative options

In CID, alternative treatment options with proven long-term efficacy and even curative potential are still limited. Autologous stem cell gene therapy may be considered as an experimental option in WAS [74]. Stem cell or somatic cell gene therapy for other forms of CID are in developmental stage and may become available in clinical trials in the next few years [15].

### Chronic granulomatous disease and other phagocyte disorders

#### Background

HSCT is the only well established cure for neutrophil disorders such as chronic granulomatous disease (CGD) and leukocyte adhesion deficiency (LAD). Outcome of HSCT for CGD is superior to conservative management [75]. A recent IEWP study reported excellent overall and event-free survival in CGD, particularly when transplants were performed with matched donors and at younger age [16]. In a recent international study in LAD, excellent survival was reported in matched donor HSCT [10]. As in CGD, insufficiently controlled inflammation pretransplant may have an unfavorable impact on the risk for graft failure and aGVHD [76].

#### Regimens

C and D are the preferred regimens as reduced toxicity conditioning leads to sustained neutrophil production of donor origin in the majority of transplanted patients, and stable mixed chimerism (>20% myeloid) is sufficient to protect against the risk of infections. This is less clear for the inflammatory component of CGD and related disorders. Some centers may prefer regimen A or B to favor myeloid engraftment, but there is no published data to date comparing outcomes between A, B, C, and D. E may provide sufficient myeloablation in certain individuals, but expected graft failure rates are higher than with A–D, and may exceed 20%. Serotherapy by Alemtuzumab, ATLG-Grafalon, or ATG-Thymoglobuline is recommended for all CGD-patients because of extensive inflammation even when MSD donors are used. The best possible control of autoinflammation (i.e., colitis, lung disease) is recommended pre HSCT, even though complete remissions may not always be achievable.

#### Specific considerations

For haploidentical and cord blood recipients use regimen A or B to ensure good myeloid engraftment. In X-CGD, carrier family donors should generally be avoided as they may have inflammatory and autoimmune symptoms [21]. In the absence of other suitable donors, female carriers may be considered as donors after careful analysis including functional tests and X-inactivation studies, as well as DHR analysis. HSCT should be considered as early as possible prior to the onset of disease-related organ damage [16].

#### Alternative options

Autologous stem cell gene therapy as a possible alternative for HSCT is currently being evaluated in clinical trials [77].

### Osteopetrosis

#### Background

Allogenic HSCT is the therapy of choice for patients with infantile osteopetrosis, but it may also be considered in (older) patients with intermediate forms [78, 79]. Contraindications in specific subtypes must be excluded: (a) osteoclast extrinsic forms with RANKL mutations and (b) neurodegenerative forms due to mutations in OSTM1 or in CLCN7. Whereas obviously all patients with OSTM1- mutations will invariably develop severe neurodegeneration, this is the case in about 50% of patients with CLCN7 mutations. Developmental delay, failure to thrive and rather specific EEG changes are early signs of neurodegerenative disease (A. Schulz, personal communication).

#### Regimens

Regimen A is preferred since there is a high risk of graft failure in this disease and myeloablative conditioning is necessary. Regimen B is a clinical option as well, particularly in patients with advanced disease. No serotherapy is necessary for MSD, whereas it is recommended in alternative donors.

#### Specific considerations

Veno-occlusive disease (VOD) and pulmonary arterial hypertension are a concern in small infants, in whom prophylaxis may be considered. For haplo transplants, the T replete approach is now recommended (see Table 3), since this protocol leads to more robust engraftment [25] (D. Moshous and A. Schulz, personal communication). Because of the high risk of disease specific side effects in osteopetrosis transplants, HSCT in osteopetrosis should be performed in experienced centers only, in particular when using haploidentical donors.

#### Alternative options

Gene therapy is explored in preclinical experiments only so far but may progress to trial in the coming years. We do not recommend long term steroids as conservative treatment because of limited efficacy and considerable side effects.

### Hemophagocytic lymphohistiocytosis (HLH)

#### Background

Regardless of the underlying genetic cause of primary HLH, disease remission at the time of HSCT remains a key factor in overall survival. The challenge is often to balance achieving disease control and reaching HSCT in a timely manner [80]. Highly immuno- and myelosuppresive drugs are used to control HLH prior to HSCT and these are associated with multi-organ toxicity and infective complications. More targeted novel therapies including alemtuzumab may reduce this toxicity and improve the patients’ condition prior to HSCT [81]. The efficacy of anti IFN gamma antibodies [82, 83] and JAK-Inhibitors need yet to be evaluated in prospective studies. VOD is common in this group of patients, mainly due to treatment related toxicity and disease, and therefore we suggest the use of prophylactic defibrotide in infants under 18 months of age or those over 18 months with clear hepatic involvement.

Historically, outcomes post HSCT were poor when using a fully myeloablative regimen but have significantly improved for most primary HLH disorders with the advent of reduced toxicity conditioning protocols and better HLH control prior to conditioning [9, 80]. Stable high-level donor chimerism is desired and mixed chimerism is seen more commonly after treosulfan and melphalan based regimens. It is likely that >20–30% T-cell chimerism is sufficient to protect against disease relapse [84].

XIAP deficient patients warrant specific mention here, as the reported outcome for this cohort is significantly worse than for other forms of primary HLH even in the context of RIC regimes. These patients are particularly sensitive to alkylating agents and appear to suffer from more severe GvHD which leads to higher mortality. However, if HLH is in remission and a reduced regimen is used, survival after HSCT is reported to be between 86 and 100% [45, 85]. The associated colitis can take a long period post-HSCT to resolve and HLH reactivation has been reported in one series to occur in up to 50% of patients [85].

#### Regimens

Conditioning: A, B, C, and D.

#### Specific considerations

Serotherapy may be adapted depending on whether serotherapy was given as prior HLH therapy. Serotherapy should also be considered as part of the conditioning to control disease if not in complete remission, even in the genoidentical setting.

### Primary immune dysregulation disorders (PIRDs)

#### Background

An increasing number of diseases of immune dysregulation are emerging which may be amenable to treatment with HSCT [13, 86]. These include T regulatory cell defects such as IPEX syndrome and CTLA4 deficiency and immune dysregulation with colitis. A large multicenter study of patients with IPEX syndrome showed a clear advantage in overall survival and quality of life in transplanted patients compared to those treated with immunosuppression such that all patients with this disease should be considered for transplant [87]. Mixed chimerism is sufficient for cure and in patients with mixed chimerism, donor chimerism in T regulatory cells has been shown to be higher than in other cell lines. No significant outcome difference according to conditioning, donor type and age at transplant has been shown, so fully myeloablative conditioning may not be required in all PIRDs possibly with the exception of gain-of-function diseases. An increasing number of reports are published for other disorders including but not restricted to CTLA4 [88, 89], LRBA [90, 91], ZAP70 [92, 93], and STAT1 gain-of-function [94].

#### Regimens

Reduced toxicity regimens are preferred, but depending on the clinical condition and co-morbidity protocols A, B, C, or D may be used. Data on the relevance of donor chimerism for cure are still limited, however in case of gain-of-function diseases and when aiming for complete donor chimerism, protocol A or B is probably preferred.

#### Specific considerations

Severity of disease at transplant is the most important predictor of success and immunosuppression prior to transplant to control the inflammatory features is of paramount importance. It can be especially challenging to decide if and when to transplant in these patients. Transplant outcome is better before organ damage and in the absence of ongoing severe inflammation. Targeted biological agents such as abatacept or ruxolitinib are increasingly available and can result in significant reduction in disease activity, but the complications of long-term use of these agents could be significant. Use of these agents as a bridging therapy to optimize condition of the patient prior to transplant is recommended [86]. Molecular diagnosis is important particulary in Very-early-onset Inflammatory Bowel Disease, as HSCT is not indicated for an enteropathy due to an epithelial defect [95, 96]. Given the highly variable genotype–phenotype correlation in most of these diseases, family donors should be screened to avoid using an affected donor who may have a mild phenotype or late onset disease.

#### Alternative options

Gene therapy trials for a number of these disorders are in preclinical status.

### Adolescent and young adult (AYA) population

#### Background

Adolescent and adult patients (>15 years) with IEI are increasingly being referred for consideration of HSCT. For many IEI patients, complications accumulate with age, which can result in end organ damage, reduced quality of life and early death. However, identifying which patients and when may benefit from HSCT remains challenging, in part due to phenotypic heterogeneity and absence of a genetic diagnosis in many.

Nonetheless, advances in genetic diagnostics, improved survival into adulthood with conservative treatment and recent data demonstrating good outcomes following HSCT in these older patients have led to this change in clinical practice. Both single center and multi-center studies have shown encouraging results in this age group [16, 46, 73, 97], including patients with a degree of preexisting organ damage, infectious burden or malignancy at the time of HSCT, which in some studies have previously resulted in worse outcome [98, 99].

#### Preferred regimen and specific considerations

In older patients and those with higher HCT-CI scores, reduced intensity conditioning regimens are preferred to limit excess toxicity (C, D, or E). Special attention should be placed on the higher risk of GVHD in this patient group compared to children. As with other IEI, the best possible control of autoimmunity and autoinflammation (i.e., colitis, lung disease) is recommended pre HSCT, even though complete remissions may not always be achievable. Pre-HSCT counseling taking into account aspects such as fertility, sexuality, social issues, and the limitation of HSCT to correct irreversible organ damage is especially challenging and important in this patient group.

#### Alternative options

For some specific forms of IEI, targeted therapies may offer a bridge to HSCT or be offered as an alternative therapy [91, 100]. Autologous stem cell gene therapy in older patients is currently being evaluated in clinical trials [15, 74, 77].

## Appendix 1Chemotherapy protocols A–F

**Table:**

	Days to HSCT	−7	−6	−5	−4	−3	−2	−1	0
Protocol									
**A**									
Busulfan (AUC 85–95)^a^				x	x	x	x		
Fludarabine (4 × 40 mg/m^2^)				x	x	x	x		
**B**									
Treosulfan (3 × 10-14 g/m^2^)^c^				x	x	x			
Fludarabine (5 × 30 mg/m^2^)			x	x	x	x	x		
Thiotepa (1×8/2×5 mg/kg)			x						
**C**									
Busulfan (AUC 60-70)^b^				x	x	x	(x)		
Fludarabine (5-6×30 mg/m^2^)		(x)	x	x	x	x	x		
**D**									
Treosulfan (3×10-14 g/m^2^)^c^				x	x	x			
Fludarabine (5×30 mg/m^2^)			x	x	x	x	x		
**E**									
Melphalan (1×140/2×70 mg/m^2^)							x		
Fludarabine (5×30 mg/m^2^))			x	x	x	x	x		
**F**									
Cyclophosphamide (4×5 mg/kg)				x	x	x	x		
Fludarabine (5×30 mg/kg)			x	x	x	x	x		

^a^see Table 2 for dosing scheme. If necessary for TDM, busulfan (and fludarabine) administration may be altered to day -6 till -3.

^b^see Table 2 for dosing scheme. Total dose may be administered in 3–4 days.

^c^3 × 10 g/m^2^ in < 0.5m^2^ body surface area, 3 × 12 g/m^2^ in 0.5-1.0 m^2^, 3 × 14 g/m^2^ in ≥1 m^2^

Note: Serotherapy is recommended in all alternative donor transplants and may be considered when using HLA matched siblings (see paragraph “ATG/ATLG and alemtuzumab” for further details). The recommended standard dose ranges for unmanipulated matched grafts are: Thymoglobulin 5-10 mg/kg (total dose, in 2-4 days), Grafalon 15-30 mg/kg (total dose, in 3 days) and alemtuzumab 0.6-1.0 mg/kg (total dose, in 3-5 days), starting at day -8/7. In specific cases, individualized adaptations may be required, preferably based on TDM. Specific recommendations are provided for the two haplo-protocols (Table 3).

## References

[1] Bousfiha A, Jeddane L, Picard C, Al-Herz W, Ailal F, Chatila T (2020). Human inborn errors of immunity: 2019 update of the IUIS phenotypical classification. J Clin Immunol.

[2] Gennery AR, Slatter MA, Grandin L, Taupin P, Cant AJ, Veys P (2010). Transplantation of hematopoietic stem cells and long-term survival for primary immunodeficiencies in Europe: entering a new century, do we do better?. J Allergy Clin Immunol.

[3] Ferrua F, Galimberti S, Courteille V, Slatter MA, Booth C, Moshous D (2019). Hematopoietic stem cell transplantation for CD40 ligand deficiency: results from an EBMT/ESID-IEWP-SCETIDE-PIDTC study. J Allergy Clin Immunol.

[4] Chiesa R, Wang J, Blok H-J, Hazelaar S, Neven B, Moshous D, et al. Haematopoietic cell transplantation in chronic granulomatous disease: a study on 712 children and adults. *Blood.* 2020. 10.1182/blood.2020005590. 10.1182/blood.2020005590 32614953

[5] Wynn R, Schulz A. Inborn errors of metabolism and osteopetrosis. In: th, Carreras E, Dufour C, Mohty M, Kroger N(eds). The EBMT handbook: hematopoietic stem cell transplantation and cellular therapies: Cham (CH), 2019, pp 671–6.

[6] Albert M, Lankester A, Gennery A. Primary immunodeficiencies. In: th, Carreras E, Dufour C, Mohty M, Kroger N (eds). The EBMT handbook: hematopoietic stem cell transplantation and cellular therapies: Cham (CH), 2019, pp 663-70. 32091673

[7] Aydin SE, Freeman AF, Al-Herz W, Al-Mousa HA, Arnaout RK, Aydin RC (2019). Hematopoietic stem cell transplantation as treatment for patients with DOCK8 deficiency. J Allergy Clin Immunol Pract.

[8] Ghosh S, Kostel Bal S, Edwards ESJ, Pillay B, Jimenez-Heredia R, Rao G, et al. Extended clinical and immunological phenotype and transplant outcome in CD27 and CD70 deficiency. *Blood*. 2020. 10.1182/blood.2020006738 10.1182/blood.2020006738 PMC7735164 32603431

[9] Felber M, Steward CG, Kentouche K, Fasth A, Wynn RF, Zeilhofer U (2020). Targeted busulfan-based reduced-intensity conditioning and HLA-matched HSCT cure hemophagocytic lymphohistiocytosis. Blood Adv.

[10] Bakhtiar S, Salzmann-Manrique E, Blok HJ, Eikema DJ, Hazelaar S, Ayas M (2021). Allogeneic hematopoietic stem cell transplantation in leukocyte adhesion deficiency type I and III. Blood Adv.

[11] Fischer A, Provot J, Jais JP, Alcais A, Mahlaoui N, members of the CFPIDsg. (2017). Autoimmune and inflammatory manifestations occur frequently in patients with primary immunodeficiencies. J Allergy Clin Immunol.

[12] Tangye SG, Al-Herz W, Bousfiha A, Chatila T, Cunningham-Rundles C, Etzioni A (2020). Human inborn errors of immunity: 2019 update on the classification from the International Union of Immunological Societies Expert Committee. J Clin Immunol.

[13] Chan AY, Leiding JW, Liu X, Logan BR, Burroughs LM, Allenspach EJ (2020). Hematopoietic cell transplantation in patients with primary immune regulatory disorders (PIRD): a Primary Immune Deficiency Treatment Consortium (PIDTC) survey. Front Immunol.

[14] Kohn DB, Booth C, Shaw KL, Xu-Bayford J, Garabedian E, Trevisan V (2021). Autologous ex vivo lentiviral gene therapy for adenosine deaminase deficiency. NEJM.

[15] Staal FJT, Aiuti A, Cavazzana M (2019). Autologous stem-cell-based gene therapy for inherited disorders: state of the art and perspectives. Front Pediatr.

[16] Chiesa R, Wang J, Blok HJ, Hazelaar S, Neven B, Moshous D (2020). Hematopoietic cell transplantation in chronic granulomatous disease: a study of 712 children and adults. Blood.

[17] Slatter MA, Rao K, Abd Hamid IJ, Nademi Z, Chiesa R, Elfeky R (2018). Treosulfan and fludarabine conditioning for hematopoietic stem cell transplantation in children with primary immunodeficiency: UK experience. Biol Blood Marrow Transpl.

[18] Slatter MA, Boztug H, Potschger U, Sykora KW, Lankester A, Yaniv I (2015). Treosulfan-based conditioning regimens for allogeneic haematopoietic stem cell transplantation in children with non-malignant diseases. Bone Marrow Transpl.

[19] Gungor T, Teira P, Slatter M, Stussi G, Stepensky P, Moshous D (2014). Reduced-intensity conditioning and HLA-matched haemopoietic stem-cell transplantation in patients with chronic granulomatous disease: a prospective multicentre study. Lancet.

[20] Bartelink IH, Lalmohamed A, van Reij EM, Dvorak CC, Savic RM, Zwaveling J (2016). Association of busulfan exposure with survival and toxicity after haemopoietic cell transplantation in children and young adults: a multicentre, retrospective cohort analysis. Lancet Haematol.

[21] Battersby AC, Braggins H, Pearce MS, Cale CM, Burns SO, Hackett S (2017). Inflammatory and autoimmune manifestations in X-linked carriers of chronic granulomatous disease in the United Kingdom. J Allergy Clin Immunol.

[22] Marciano BE, Zerbe CS, Falcone EL, Ding L, DeRavin SS, Daub J (2018). X-linked carriers of chronic granulomatous disease: Illness, lyonization, and stability. J Allergy Clin Immunol.

[23] Schuetz C, Pannicke U, Jacobsen EM, Burggraf S, Albert MH, Honig M (2014). Lesson from hypomorphic recombination-activating gene (RAG) mutations: why asymptomatic siblings should also be tested. J Allergy Clin Immunol.

[24] Kurzay M, Hauck F, Schmid I, Wiebking V, Eichinger A, Jung E (2019). T-cell replete haploidentical bone marrow transplantation and post-transplant cyclophosphamide for patients with inborn errors. Haematologica.

[25] Neven B, Diana JS, Castelle M, Magnani A, Rosain J, Touzot F (2019). Haploidentical hematopoietic stem cell transplantation with post-transplant cyclophosphamide for primary immunodeficiencies and inherited disorders in children. Biol Blood Marrow Transpl.

[26] Balashov D, Shcherbina A, Maschan M, Trakhtman P, Skvortsova Y, Shelikhova L (2015). Single-center experience of unrelated and haploidentical stem cell transplantation with TCR alphabeta and CD19 depletion in children with primary immunodeficiency syndromes. Biol Blood Marrow Transpl.

[27] Shah RM, Elfeky R, Nademi Z, Qasim W, Amrolia P, Chiesa R (2018). T-cell receptor alphabeta(+) and CD19(+) cell-depleted haploidentical and mismatched hematopoietic stem cell transplantation in primary immune deficiency. J Allergy Clin Immunol.

[28] Savic RM, Cowan MJ, Dvorak CC, Pai SY, Pereira L, Bartelink IH (2013). Effect of weight and maturation on busulfan clearance in infants and small children undergoing hematopoietic cell transplantation. Biol Blood Marrow Transpl.

[29] Shukla P, Goswami S, Keizer RJ, Winger BA, Kharbanda S, Dvorak CC (2020). Assessment of a model-informed precision dosing platform use in routine clinical care for personalized busulfan therapy in the pediatric Hematopoietic Cell Transplantation (HCT) population. Front Pharm.

[30] Morillo-Gutierrez B, Beier R, Rao K, Burroughs L, Schulz A, Ewins AM (2016). Treosulfan-based conditioning for allogeneic HSCT in children with chronic granulomatous disease: a multicenter experience. Blood.

[31] Faraci M, Diesch T, Labopin M, Dalissier A, Lankester A, Gennery A (2019). Gonadal function after busulfan compared with treosulfan in children and adolescents undergoing allogeneic hematopoietic stem cell transplant. Biol Blood Marrow Transpl.

[32] Leiper A, Houwing M, Davies EG, Rao K, Burns S, Morris E (2020). Anti-Mullerian hormone and Inhibin B after stem cell transplant in childhood: a comparison of myeloablative, reduced intensity and treosulfan-based chemotherapy regimens. Bone Marrow Transpl.

[33] van der Stoep M, Bertaina A, Ten Brink MH, Bredius RG, Smiers FJ, Wanders DCM (2017). High interpatient variability of treosulfan exposure is associated with early toxicity in paediatric HSCT: a prospective multicentre study. Br J Haematol.

[34] Chiesa R, Standing JF, Winter R, Nademi Z, Chu J, Pinner D (2020). Proposed therapeutic range of treosulfan in reduced toxicity pediatric allogeneic hematopoietic stem cell transplant conditioning: results from a prospective trial. Clin Pharm Ther.

[35] Mohanan E, Panetta JC, Lakshmi KM, Edison ES, Korula A, Fouzia NA (2017). Population pharmacokinetics of fludarabine in patients with aplastic anemia and Fanconi anemia undergoing allogeneic hematopoietic stem cell transplantation. Bone Marrow Transpl.

[36] Ivaturi V, Dvorak CC, Chan D, Liu T, Cowan MJ, Wahlstrom J (2017). Pharmacokinetics and model-based dosing to optimize fludarabine therapy in pediatric hematopoietic cell transplant recipients. Biol Blood Marrow Transpl.

[37] Chung H, Hong KT, Lee JW, Rhee SJ, Kim S, Yoon SH (2019). Pharmacokinetics of fludarabine and its association with clinical outcomes in paediatric haematopoietic stem cell transplantation patients. Bone Marrow Transpl.

[38] Lehmberg K, Albert MH, Beier R, Beutel K, Gruhn B, Kroger N (2014). Treosulfan-based conditioning regimen for children and adolescents with hemophagocytic lymphohistiocytosis. Haematologica.

[39] Naik S, Eckstein O, Sasa G, Heslop HE, Krance RA, Allen C (2020). Incorporation of thiotepa in a reduced intensity conditioning regimen may improve engraftment after transplant for HLH. Br J Haematol.

[40] Slack J, Albert MH, Balashov D, Belohradsky BH, Bertaina A, Bleesing J (2018). Outcome of hematopoietic cell transplantation for DNA double-strand break repair disorders. J Allergy Clin Immunol.

[41] Wolska-Kusnierz B, Gennery AR (2019). Hematopoietic stem cell transplantation for dna double strand breakage repair disorders. Front Pediatr.

[42] Rao K, Amrolia PJ, Jones A, Cale CM, Naik P, King D (2005). Improved survival after unrelated donor bone marrow transplantation in children with primary immunodeficiency using a reduced-intensity conditioning regimen. Blood.

[43] Ritchie DS, Seymour JF, Roberts AW, Szer J, Grigg AP (2001). Acute left ventricular failure following melphalan and fludarabine conditioning. Bone Marrow Transpl.

[44] Allen CE, Marsh R, Dawson P, Bollard CM, Shenoy S, Roehrs P (2018). Reduced-intensity conditioning for hematopoietic cell transplant for HLH and primary immune deficiencies. Blood.

[45] Marsh RA, Rao K, Satwani P, Lehmberg K, Muller I, Li D (2013). Allogeneic hematopoietic cell transplantation for XIAP deficiency: an international survey reveals poor outcomes. Blood.

[46] Fox TA, Chakraverty R, Burns S, Carpenter B, Thomson K, Lowe D (2018). Successful outcome following allogeneic hematopoietic stem cell transplantation in adults with primary immunodeficiency. Blood.

[47] Willemsen L, Jol-van der Zijde CM, Admiraal R, Putter H, Jansen-Hoogendijk AM, Ostaijen-Ten Dam MM (2015). impact of serotherapy on immune reconstitution and survival outcomes after stem cell transplantations in children: thymoglobulin versus alemtuzumab. Biol Blood Marrow Transpl.

[48] Admiraal R, van Kesteren C, Jol-van der Zijde CM, Lankester AC, Bierings MB, Egberts TC (2015). Association between anti-thymocyte globulin exposure and CD4 + immune reconstitution in paediatric haemopoietic cell transplantation: a multicentre, retrospective pharmacodynamic cohort analysis. Lancet Haematol.

[49] Oostenbrink LVE, Jol-van der Zijde CM, Kielsen K, Jansen-Hoogendijk AM, Ifversen M, Muller KG (2019). Differential elimination of anti-thymocyte globulin of fresenius and genzyme impacts T-cell reconstitution after hematopoietic stem cell transplantation. Front Immunol.

[50] Bonifazi F, Rubio MT, Bacigalupo A, Boelens JJ, Finke J, Greinix H (2020). Rabbit ATG/ATLG in preventing graft-versus-host disease after allogeneic stem cell transplantation: consensus-based recommendations by an international expert panel. Bone Marrow Transpl.

[51] Admiraal R, Lindemans CA, van Kesteren C, Bierings MB, Versluijs AB, Nierkens S (2016). Excellent T-cell reconstitution and survival depend on low ATG exposure after pediatric cord blood transplantation. Blood.

[52] Czerw T, Labopin M, Schmid C, Cornelissen JJ, Chevallier P, Blaise D (2016). High CD3 + and CD34 + peripheral blood stem cell grafts content is associated with increased risk of graft-versus-host disease without beneficial effect on disease control after reduced-intensity conditioning allogeneic transplantation from matched unrelated donors for acute myeloid leukemia - an analysis from the Acute Leukemia Working Party of the European Society for Blood and Marrow Transplantation. Oncotarget.

[53] Hough R, Danby R, Russell N, Marks D, Veys P, Shaw B (2016). Recommendations for a standard UK approach to incorporating umbilical cord blood into clinical transplantation practice: an update on cord blood unit selection, donor selection algorithms and conditioning protocols. Br J Haematol.

[54] Bertaina A, Merli P, Rutella S, Pagliara D, Bernardo ME, Masetti R (2014). HLA-haploidentical stem cell transplantation after removal of alphabeta+ T and B cells in children with nonmalignant disorders. Blood.

[55] Klein OR, Bapty S, Lederman HM, Younger MEM, Zambidis ET, Jones RJ (2021). Reduced intensity bone marrow transplantation with post-transplant cyclophosphamide for pediatric inherited immune deficiencies and bone marrow failure syndromes. J Clin Immunol.

[56] Fernandes JF, Bonfim C, Kerbauy FR, Rodrigues M, Esteves I, Silva NH (2018). Haploidentical bone marrow transplantation with post transplant cyclophosphamide for patients with X-linked adrenoleukodystrophy: a suitable choice in an urgent situation. Bone Marrow Transpl.

[57] Bernatowska EA, Wolska-Kusnierz B, Pac M, Kurenko-Deptuch M, Zwolska Z, Casanova JL (2007). Disseminated bacillus Calmette-Guerin infection and immunodeficiency. Emerg Infect Dis.

[58] Fernandes JF, Nichele S, Arcuri LJ, Ribeiro L, Zamperlini-Netto G, Loth G (2020). Outcomes after haploidentical stem cell transplantation with post-transplantation cyclophosphamide in patients with primary immunodeficiency diseases. Biol Blood Marrow Transpl.

[59] Haddad E, Hoenig M (2019). Hematopoietic stem cell transplantation for Severe Combined Immunodeficiency (SCID). Front Pediatr.

[60] Schuetz C, Neven B, Dvorak CC, Leroy S, Ege MJ, Pannicke U (2014). SCID patients with ARTEMIS vs RAG deficiencies following HCT: increased risk of late toxicity in ARTEMIS-deficient SCID. Blood.

[61] Kohn DB, Hershfield MS, Puck JM, Aiuti A, Blincoe A, Gaspar HB (2019). Consensus approach for the management of severe combined immune deficiency caused by adenosine deaminase deficiency. J Allergy Clin Immunol.

[62] Hoenig M, Lagresle-Peyrou C, Pannicke U, Notarangelo LD, Porta F, Gennery AR (2017). Reticular dysgenesis: international survey on clinical presentation, transplantation, and outcome. Blood.

[63] Dvorak CC, Hassan A, Slatter MA, Honig M, Lankester AC, Buckley RH (2014). Comparison of outcomes of hematopoietic stem cell transplantation without chemotherapy conditioning by using matched sibling and unrelated donors for treatment of severe combined immunodeficiency. J Allergy Clin Immunol.

[64] Villa A, Notarangelo LD, Roifman CM (2008). Omenn syndrome: inflammation in leaky severe combined immunodeficiency. J Allergy Clin Immunol.

[65] Villa A, Notarangelo LD (2019). RAG gene defects at the verge of immunodeficiency and immune dysregulation. Immunol Rev.

[66] Riballo E, Critchlow SE, Teo SH, Doherty AJ, Priestley A, Broughton B (1999). Identification of a defect in DNA ligase IV in a radiosensitive leukaemia patient. Curr Biol.

[67] Albert MH, Freeman AF (2019). Wiskott-Aldrich Syndrome (WAS) and dedicator of cytokinesis 8- (DOCK8) deficiency. Front Pediatr.

[68] Petersheim D, Massaad MJ, Lee S, Scarselli A, Cancrini C, Moriya K (2018). Mechanisms of genotype-phenotype correlation in autosomal dominant anhidrotic ectodermal dysplasia with immune deficiency. J Allergy Clin Immunol.

[69] Lum SH, Neven B, Slatter MA, Gennery AR (2019). Hematopoietic cell transplantation for MHC class II deficiency. Front Pediatr.

[70] Speckmann C, Doerken S, Aiuti A, Albert MH, Al-Herz W, Allende LM (2017). A prospective study on the natural history of patients with profound combined immunodeficiency: an interim analysis. J Allergy Clin Immunol.

[71] Neven B, Ferrua F (2019). Hematopoietic stem cell transplantation for combined immunodeficiencies, on behalf of IEWP-EBMT. Front Pediatr.

[72] Burroughs LM, Petrovic A, Brazauskas R, Liu X, Griffith LM, Ochs HD (2020). Excellent outcomes following hematopoietic cell transplantation for Wiskott-Aldrich syndrome: a PIDTC report. Blood.

[73] Albert MH, Hauck F, Wiebking V, Aydin S, Notheis G, Koletzko S (2018). Allogeneic stem cell transplantation in adolescents and young adults with primary immunodeficiencies. J Allergy Clin Immunol Pract.

[74] Morris EC, Fox T, Chakraverty R, Tendeiro R, Snell K, Rivat C (2017). Gene therapy for Wiskott-Aldrich syndrome in a severely affected adult. Blood.

[75] Gungor T, Chiesa R (2020). Cellular therapies in chronic granulomatous disease. Front Pediatr.

[76] Bakhtiar S, Shadur B, Stepensky P (2019). The evidence for allogeneic hematopoietic stem cell transplantation for congenital neutrophil disorders: a comprehensive review by the Inborn Errors Working Party Group of the EBMT. Front Pediatr.

[77] Kohn DB, Booth C, Kang EM, Pai SY, Shaw KL, Santilli G (2020). Lentiviral gene therapy for X-linked chronic granulomatous disease. Nat Med.

[78] Natsheh J, Drozdinsky G, Simanovsky N, Lamdan R, Erlich O, Gorelik N (2016). Improved outcomes of hematopoietic stem cell transplantation in patients with infantile malignant osteopetrosis using fludarabine-based conditioning. Pediatr Blood Cancer.

[79] Stepensky P, Grisariu S, Avni B, Zaidman I, Shadur B, Elpeleg O (2019). Stem cell transplantation for osteopetrosis in patients beyond the age of 5 years. Blood Adv.

[80] Lehmberg K, Moshous D, Booth C (2019). Haematopoietic stem cell transplantation for primary haemophagocytic lymphohistiocytosis. Front Pediatr.

[81] Moshous D, Briand C, Castelle M, Dupic L, Morelle G, Abou Chahla W, et al. Alemtuzumab as first line treatment in children with familial lymphohistiocytosis. *Blood.* 2019; 134. 10.1182/blood-2019-124477

[82] Locatelli F, Jordan MB, Allen C, Cesaro S, Rizzari C, Rao A (2020). Emapalumab in children with primary hemophagocytic lymphohistiocytosis. N Engl J Med.

[83] Ehl S, von Bahr Greenwood T, Bergsten E, Fischer A, Henter JI, Hines M (2021). Is neutralization of IFN-gamma sufficient to control inflammation in HLH?. Pediatr Blood Cancer.

[84] Hartz B, Marsh R, Rao K, Henter JI, Jordan M, Filipovich L (2016). The minimum required level of donor chimerism in hereditary hemophagocytic lymphohistiocytosis. Blood.

[85] Ono S, Okano T, Hoshino A, Yanagimachi M, Hamamoto K, Nakazawa Y (2017). Hematopoietic stem cell transplantation for XIAP deficiency in Japan. J Clin Immunol.

[86] Bakhtiar S, Fekadu J, Seidel MG, Gambineri E (2019). Allogeneic hematopoietic stem cell transplantation for congenital immune dysregulatory disorders. Front Pediatr.

[87] Barzaghi F, Amaya Hernandez LC, Neven B, Ricci S, Kucuk ZY, Bleesing JJ (2018). Long-term follow-up of IPEX syndrome patients after different therapeutic strategies: an international multicenter retrospective study. J Allergy Clin Immunol.

[88] Schwab C, Gabrysch A, Olbrich P, Patino V, Warnatz K, Wolff D (2018). Phenotype, penetrance, and treatment of 133 cytotoxic T-lymphocyte antigen 4-insufficient subjects. J Allergy Clin Immunol.

[89] Slatter MA, Engelhardt KR, Burroughs LM, Arkwright PD, Nademi Z, Skoda-Smith S (2016). Hematopoietic stem cell transplantation for CTLA4 deficiency. J Allergy Clin Immunol.

[90] Habibi S, Zaki-Dizaji M, Rafiemanesh H, Lo B, Jamee M, Gamez-Diaz L (2019). Clinical, immunologic, and molecular spectrum of patients with lps-responsive beige-like anchor protein deficiency: a systematic review. J Allergy Clin Immunol Pr.

[91] Tesch VK, Abolhassani H, Shadur B, Zobel J, Mareika Y, Sharapova S (2020). Long-term outcome of LRBA deficiency in 76 patients after various treatment modalities as evaluated by the immune deficiency and dysregulation activity (IDDA) score. J Allergy Clin Immunol.

[92] Sharifinejad N, Jamee M, Zaki-Dizaji M, Lo B, Shaghaghi M, Mohammadi H (2020). Clinical, immunological, and genetic features in 49 patients with ZAP-70 deficiency: a systematic review. Front Immunol.

[93] Cuvelier GD, Rubin TS, Wall DA, Schroeder ML (2016). Long-term outcomes of hematopoietic stem cell transplantation for ZAP70 deficiency. J Clin Immunol.

[94] Leiding JW, Okada S, Hagin D, Abinun M, Shcherbina A, Balashov DN (2018). Hematopoietic stem cell transplantation in patients with gain-of-function signal transducer and activator of transcription 1 mutations. J Allergy Clin Immunol.

[95] Uhlig HH, Schwerd T, Koletzko S, Shah N, Kammermeier J, Elkadri A (2014). The diagnostic approach to monogenic very early onset inflammatory bowel disease. Gastroenterology.

[96] Ouahed J, Spencer E, Kotlarz D, Shouval DS, Kowalik M, Peng K (2020). Very early onset inflammatory bowel disease: a clinical approach with a focus on the role of genetics and underlying immune deficiencies. Inflamm Bowel Dis.

[97] Dimitrova D, Gea-Banacloche J, Steinberg SM, Sadler JL, Hicks SN, Carroll E (2020). Prospective study of a novel, radiation-free, reduced-intensity bone marrow transplantation platform for primary immunodeficiency diseases. Biol Blood Marrow Transpl.

[98] Morris EC, Albert MH (2019). Allogeneic HSCT in adolescents and young adults with primary immunodeficiencies. Front Pediatr.

[99] Wehr C, Gennery AR, Lindemans C, Schulz A, Hoenig M, Marks R (2015). Multicenter experience in hematopoietic stem cell transplantation for serious complications of common variable immunodeficiency. J Allergy Clin Immunol.

[100] Forbes LR, Vogel TP, Cooper MA, Castro-Wagner J, Schussler E, Weinacht KG (2018). Jakinibs for the treatment of immune dysregulation in patients with gain-of-function signal transducer and activator of transcription 1 (STAT1) or STAT3 mutations. J Allergy Clin Immunol.

[101] Im A, Rashidi A, Wang T, Hemmer M, MacMillan ML, Pidala J (2020). Risk factors for graft-versus-host disease in haploidentical hematopoietic cell transplantation using post-transplant cyclophosphamide. Biol Blood Marrow Transpl.

